# Nebivolol Attenuates Neutrophil Lymphocyte Ratio: A Marker of Subclinical Inflammation in Hypertensive Patients

**DOI:** 10.1155/2017/7643628

**Published:** 2017-07-27

**Authors:** Mazhar Hussain, Muhammad Saeed, Muhammad Zafar Majeed Babar, Moazzam Ali Atif, Lubna Akhtar

**Affiliations:** ^1^Department of Pharmacology & Therapeutics, Sheikh Zayed Medical College/Hospital, Rahim Yar Khan, Punjab, Pakistan; ^2^Department of Biochemistry, University Medical & Dental College, Faisalabad, Punjab, Pakistan; ^3^Department of Medicine, Sheikh Zayed Medical College/Hospital, Rahim Yar Khan, Punjab, Pakistan

## Abstract

**Background:**

High value of neutrophil lymphocyte ratio (NLR) is a strong independent predictor and biomarker of ongoing vascular inflammation in various cardiovascular disorders.

**Objective:**

The main focus of the study is to investigate the effect of nebivolol on NLR in mild to moderate hypertensive patients in comparison with metoprolol. In addition, BMI, blood pressure, TLC count, blood sugar, and lipid profile were also assayed before and after treatment.

**Materials and Methods:**

In this 12-week prospective double-blinded randomized study, 120 patients with mild to moderate hypertension were randomly divided into two groups to prescribed daily dose of tab nebivolol 5–10 mg and metoprolol 50–100 mg, respectively, for 12 weeks. The data were analyzed using SPSS 16 software.

**Results:**

A total of 100 patients completed the study. Both drugs lowered blood pressure significantly, nebivolol 20.5/10.5 and metoprolol 22.5/11.2 (*p* < 0.001) from baseline. Regarding inflammation, nebivolol reduced total leukocyte count (*p* = 0.005) and neutrophil count (*p* = 0.003) and increased lymphocyte count (*p* = 0.004) as compared to metoprolol. Similarly, nebivolol but not metoprolol significantly reduced NLR ratio (*p* = 0.07). Nebivolol improved lipid profile and blood sugar compared to metoprolol, but values were nonsignificant.

**Conclusion:**

Nebivolol has a strong impact on reducing NLR, a marker of subclinical inflammation in hypertensive patients. Moreover NLR can be used as a disease and drug monitoring tool in these patients.

## 1. Introduction

Hypertension affects about one-fourth of the adult population globally and this number will increase to 29% by year 2025. The prevalence of hypertension will be much higher in developing countries in upcoming years because of strong influence of urbanization and lifestyle changes to its underlying risk factors. In addition, healthcare facilities in developing world for routine screening of hypertensive patients are grossly underutilized. Approximately one-third of deaths in the world population are mainly due to cardiovascular disease with hypertension being one of its route cause. High blood pressure itself initiates a chain of events in vessels wall in the form oxidative stress, inflammation, and endothelial dysfunction that plays a fundamental role in initiation, progression, and complication of atherosclerosis in the form of cardiovascular diseases. Therefore multifaceted and multipronged strategies are needed to handle this health issue in both developed and developing nations in order to reduce economic and social disease burden [1, 2].

The role of increased leukocytes count in the pathogenesis of atherosclerosis is well understood that initiates a cascade of inflammatory reaction in the vessel wall. Increased leukocyte counts are reliable markers of systemic inflammation in clinical practice that has very strong association with coronary heart disease, cerebrovascular disease, and vascular complications associated with diabetes [3, 4]. In recent years, there was more focus on neutrophil lymphocyte ratio (NLR), which is determined in complete blood counts by simply dividing the absolute neutrophil count to absolute lymphocyte count. NLR is reliable biomarker of low grade inflammation in various clinical conditions such as diabetes, hypertension, metabolic syndrome, obesity, and lifestyle changes [5, 6]. NLR is a reliable marker of systemic inflammation and a routinely performed test because of its easy detection, availability, and cost effectiveness. It can also be used as a drug and disease monitoring tool in various diseases. Moreover, it provides prognostic as well as diagnostic information about subclinical inflammation beyond conventional risk factors. Moreover the prognosis value of NLR is comparable to C-reactive protein, tumor necrosis factor-*α*, and interleukin-1 in the detection of subclinical inflammation and endothelial dysfunction in various clinical trials [7–9].

Nebivolol is a highly cardioselective 3rd-generation long acting -1 blocker which is usually prescribed once a day as monotherapy or in combination with other antihypertensive drugs. It is only beta-blocker that has vasodilating properties and is safely prescribed in diabetics patients too as it does have any adverse effect on insulin sensitivity and serum lipid profile. It also does not cause sexual dysfunction like other beta-blockers even though it improves sexual dysfunction in diabetic patients. It also inhibits platelets aggregation, improves endothelial and left ventricular systolic dysfunction, and improves arterial stiffness, coronary and peripheral blood flow in patients of angina, myocardial infarction, chronic heart failure, and various arrhythmias [10, 11].

Apart from their beneficial effects on blood pressure, nebivolol has a very strong anti-inflammatory and antioxidant potential. Nebivolol reduces the level of various oxidative and inflammatory markers like oxidized LDL, 8-isoprostanes, CRP, interleukin- (IL-) 6, adiponectin, leptin, intercellular adhesions molecule (ICAM-1), vascular cell adhesions molecule (VCAM-1), and tumor necrosis factor-*α* in various clinical studies [12, 13]. However lab detection of these markers is costly and usually not performed on routine clinical setting. The present study was conducted to investigate the effect of nebivolol on neutrophil lymphocyte ratio (NLR) which is easily determined from routine blood test.

## 2. Patients and Methods

This twelve-week prospective randomized double-blinded clinical trial was carried out at cardiology outdoor of Sheikh Zayed Medical College/Hospital, Rahim Yar Khan, from 5 November 2016 to 5 January 2017. An ethical committee permission and written informed consent was obtained from all patients before enrollment. Initially two hundred and forty patients were recruited in cardiology outdoor unit on the basis of chief complaints of headache, dyspnea, dizziness, and insomnia. Out of which 120 patients, aged 32–56 years with mild to moderate hypertensions according to World Health Organization (WHO)/International Society of Hypertension (ISH) criteria [14], who were never treated with any hypertensive drug in past, were enrolled in the study. The exclusion criteria was all conditions which affect inflammation such as smoking, alcohol, any acute or chronic inflammatory diseases, diabetes, trauma, pregnancy, secondary hypertension, dyslipidemia, liver disorders, kidney dysfunction, ischemic heart disease, and CNS disorders. In addition, detailed history was taken about drugs which affect inflammation such as statins, NSAIDS, corticosteroids, oral contraceptive, opioids, and various immunosuppressive agents.

A randomization was done by allocation of random number in ascending order in blocks to all patients generated by computed software. All patients remained unknown about the treatment plan to each other throughout the study period. After randomization patients were divided into two equal groups to prescribe daily dose of tablet nebivolol 5–10 mg and metoprolol 50–100 mg, respectively. The dose was adjusted according to their blood pressure which was kept below 140/90 mmHg. Those patients were excluded from the study if their targeted blood pressure was not achieved in spite of dose adjustment during first two weeks of therapy.

Brachial blood pressure was measured in right arm in sitting posture by auscultatory method using standard mercury sphygmomanometer apparatus as a mean of three consecutive readings at an interval of 10 minutes. BMI was calculated by standard formula weight in kg/height in m^2^ without shoes and wearing light clothes. Blood sample was calculated from the median cubital vein after an overnight fasting of 12 hours. The samples were used for analyzing TLC count, blood sugar, lipid profile, serum total cholesterol (TC), HDL-cholesterol (HDL-C), serum triglycerides (TG), and LDL-cholesterol (LDL-C). Lipid profile and blood sugar were estimated by calibrated semiautomated analyzer. Total leukocyte count was analyzed by automated hematology analyzer Sysmex KX-21. Neutrophil lymphocyte ratio (NLR) was calculated by dividing the absolute neutrophil ratio to absolute lymphocyte ratio.

## 3. Data Analysis

All numeric data were presented as mean ± standard deviation and percentages. Continuous variables for normal distribution were assessed by using Kolmogorov-Smirnov test. With respect to these test results, we used Mann–Whitney* U* and independent* t*-test as appropriate. The comparisons between two groups in terms of statistical significance were done by chi-squared, Mann–Whitney* U* and independent* t*-test. Continuous variables were compared by two-tailed paired* t*-test by pre- and posttreatment. Data were analyzed by using SPSS 16 software. A *p* < 0.05 was considered to be statistically significant.

## 4. Results

A total of 120 patients were enrolled in the study, out of which, 10 patients, 4 in the nebivolol and 6 in metoprolol, cannot complete the study because they were lost of follow-up. Both groups tolerated the drug very well with no major adverse effects being reported during the study; however, two patients in nebivolol group and one patient in metoprolol group developed symptomatic bradycardia and withdrawal from the study. Three patients in nebivolol and four patients in metoprolol group were found to be nonresponders to drug regime and also withdraw from the study. A total of 100 patients completed the study, 51 in nebivolol and 49 in metoprolol group. There was no significant difference in both groups in terms of baseline demographic and clinical parameters (Table 1). Both drugs lowered blood pressure significantly, nebivolol 20.5/10.5 and metoprolol 22.5/11.2 with (*p* < 0.001) from baseline. In comparison with metoprolol, nebivolol caused a significant reduction in total leukocyte count (*p* = 0.005) and neutrophil count (*p* = 0.003) and increased lymphocyte count (*p* = 0.004). Similarly, nebivolol but not metoprolol significantly reduced NLR ratio (*p* = 0.07). Nebivolol in comparison with metoprolol also improved blood sugar and lipid profile but values were found to be nonsignificant (Table 2).

## 5. Discussion

The present study was conducted to investigate the effect of nebivolol on NLR, a subclinical inflammatory marker in hypertensive patients. Subclinical inflammation is a silent killer in hypertensive patients which cannot manifest clinically for years but it can cause endothelial dysfunction that predispose to atherosclerosis and its related complication in the form of various cardiovascular and cerebrovascular disorders in future [15].

NLR is a new addition in the list of inflammatory markers and has got special attention in recent years because it has strong independent diagnostic as well as prognostic value for atherosclerosis related vascular diseases and its associated risk factors such as hypertension, diabetes, metabolic syndrome, and dyslipidemia [16]. Studies have shown the important role of NLR in hypertension. Increased NLR has strong independent association with the severity of hypertension in untreated cases of essential hypertension. Similarly NLR is a strong predictor of both resistant and nondipper hypertension in various clinical studies. Similarly a large-scale epidemiological study reveals that elevated level NLR have significant correlation with an increasing risk of developing hypertension over a follow-up period of 6 years [17–20]. In addition NLR has prognostic significance in hypertension associated complications such as heart failure, coronary artery, cerebral artery, and peripheral artery disease. In order to detect this inflammation, neutrophil to lymphocyte ratio NLR gives valuable information about the severity of atherosclerosis in various studies. This test is simply performed by dividing the absolute neutrophil count to absolute lymphocyte count in peripheral blood sample. Moreover this test is quite inexpensive, usually not requiring a proper setup and can be used as screening and drug monitoring tool in population on large scale [16, 21].

In this study, nebivolol caused a significant reduction in NLR after 12-week treatment. Nebivolol has a strong potential to reverse hypertension associated inflammatory changes by a variety of suspected mechanisms. First nebivolol improves endothelial dysfunction that causes endothelial cells to initiate inflammation by the generation of reactive oxygen species and proinflammatory cytokines [22]. Second nebivolol increases the level of nitric oxide in vessel causing vasodilatation by interacting with L-arginine/nitric oxide pathway as inflammation blocks the ability of endothelium to generate nitric oxide and prostacyclin [23]. Finally nebivolol itself has strong antioxidant and anti-inflammatory potential by reducing the production of hs-CRP, interleukin-6 (IL-6), vascular adhesion molecule (VCAM-1), adiponectin, and leptin in hypertensive patients in various clinical studies [12–24].

Regarding antihypertensive drugs, a very limited work has been done in the past to see their effect on NLR. A study conducted by Karaman et al. [25] revealed that amlodipine (calcium channel blocker) and valsartan (angiotension receptor antagonist) improved endothelial dysfunction and vascular inflammation by decreasing the level of both Von Willebrand factor and NLR in newly diagnosed hypertensive patients, while similar type of work conducted by Fici et al. [26] demonstrated that nebivolol significantly reduced red cell distribution width (RDW), total leukocyte count, and N/L ratio as compared to metoprolol in newly diagnosed hypertensive patients over a period of 6 months. Our study showed similar results to that of Fici et al. in terms of TLC count and N/L ratio but the duration of study was 3 months.

Most of the beta-blockers have adverse effect on glucose and lipid metabolisms. In this study nebivolol did not disturb lipid and glucose metabolism. Although it improved lipid profile to some extent in comparison with metoprolol but values were found to be nonsignificant. This has additional beneficial effect as both deranged blood sugar and lipid profiles themselves initiate a process of vascular inflammation. Seeing these beneficial effects of nebivolol, it should be considered in hypertensive patients in order to prevent its future complications. Moreover, NLR, a routine test, can be used as a drug and disease monitoring tool in these patients as it has diagnostic as well as prognostic value in various cardiovascular disorders.

## 6. Conclusion

Nebivolol has a strong potential to decrease NLR reverse subclinical inflammation in hypertensive patients.

## Figures and Tables

**Table 1:** Comparison of baseline characteristics and inflammatory parameters between two groups.

Parameters	Nebivolol group (*n* = 51)	Metoprolol group (*n* = 49)	*p* value^*∗∗*^
Age (years)	52.46 ± 8.32	54.60 ± 7.82	0.20^*∗∗*^
Male (*n* = %)	35 (68.6)	32 (65.3 )	0.74^*∗∗∗*^
BMI (kg/m^2^)			
Before	26.95 ± 4.82	27.24 ± 3.86	0.99^*∗∗*^
After	26.25 ± 3.56	27.45 ± 4.25	0.94^*∗∗*^
*p*	0.38^*∗*^	0.59^*∗*^	
SBP (mmHg)			
Before	149 ± 15	148 ± 14	0.36^*∗∗*^
After	131 ± 11	136 ± 8	0.68^*∗∗*^
*p*	<0.001^*∗*^	<0.001^*∗*^	
DBP (mmHg)			
Before	92 ± 10	93 ± 11	0.09^*∗∗*^
After	80 ± 11	83 ± 10	0.17^*∗∗*^
*p*	<0.001^*∗*^	<0.001^*∗*^	
WBC count × 10^9^/L			
Before	9.4 ± 3.8	9.2 ± 3.6	0.66^*∗∗*^
After	7.6 ± 3.1	9.3 ± 4.72	0.001^*∗∗∗*^
*p*	0.005^*∗*^	0.69^*∗∗*^	
Neutrophil count × 10^9^/L			
Before	7.5 ± 2.8	7.6 ± 3.2	0.26^*∗∗*^
After	4.9 ± 3.2	7.4 ± 4.5	0.009^*∗∗*^
*p*	0.003^*∗*^	0.69^*∗*^	
Lymphocyte count × 10^9^/L			
Before	2.1 ± 0.7	2.2 ± 1.62	0.72^*∗∗*^
After	2.4 ± 0.8	2.1 ± 1.45	0.02^*∗∗*^
*p*	0.004^*∗*^	0.78^*∗*^	
NLR			
Before	4.4 ± 2.2	4.5 ± 3.2	0.76^*∗∗*^
After	2.2 ± 1.5	4.4 ± 2.7	0.004^*∗∗∗*^
*p*	0.007^*∗*^	0.49^*∗*^	

Values are mean ± SD;^*∗*^paired *t*-test; ^*∗∗*^independent sample *t*-test; ^*∗∗∗*^Mann–Whitney *U* test.

**Table 2:** Comparison of the lipid profile and blood sugar between two groups.

Parameters	Nebivolol group (*n* = 51)	Metoprolol group (*n* = 49)	*p* value^*∗∗*^
Total cholesterol (mg/dl)			
Before	187.32 ± 31.22	188.82 ± 33.43	0.88
After	184.65 ± 27.35	190.68 ± 36.92	0.62
*p*^*∗*^	0.54	0.16	
Triglycerides (mg/dl)			
Before	130.36 ± 60.54	133.34 ± 43.86	0.73
After	126.45 ± 54.62	138 ± 52.45	0.62
*p*^*∗*^	0.13	0.17	
LDL-cholesterol (mg/dl)			
Before	127.42 ± 44.43	134.54 ± 42.56	0.79
After	123.65 ± 38.85	136.82 ± 36.78	0.80
*p*^*∗*^	0.54	0.82	
HDL-cholesterol (mg/dl)			
Before	43.85 ± 12.46	44.42 ± 8.62	0.64
After	45.32 ± 11.52	43.67 ± 11.82	0.49
*p*^*∗*^	0.13	0.17	
Blood sugar (mg/dl)			
Before	84.82 ± 8.82	86.42 ± 11.2	0.54
After	82.54 ± 9.89	87.56 ± 10.5	0.68
*p*^*∗*^	0.34	0.56	

LDL-C, low-density lipoprotein cholesterol; HDL-C, high-density lipoprotein cholesterol; values are mean ± SD; ^*∗*^paired *t*-test; ^*∗∗*^independent sample *t*-test.

## Additional Points

*Clinical Implication*. Elevated NLR is very important marker and has an important role in the detection of ongoing vascular inflammation. Treatment with nebivolol may improve cardiovascular outcome by decreasing NLR.* Limitation of Study*. The main limitation of the study was that NLR was not compared with other important inflammatory markers such as C-reactive protein which is the most studied marker to detect inflammation in various cardiovascular disorders. Further studies with large sample size should be conducted to compare the effect of nebivolol on various inflammatory markers.
